# Genome-wide SNP analysis reveals genetic diversity and structure of wild and cultivated olives (*Olea europaea* L.) in Oman

**DOI:** 10.1038/s41598-026-40849-0

**Published:** 2026-03-01

**Authors:** Rashid A. Al-Yahyai, Boshra Ahmed Halo, Ali M. Al-Subhi, Mahmoud W. Yaish

**Affiliations:** 1 Department of Plant Sciences, College of Agricultural and Marine Sciences, Sultan Qaboos University, Al-Khoud 123, P.O. Box 34, Muscat, Oman; 2 Department of Biology, College of Sciences, Sultan Qaboos University, Al-Khoud 123, P.O. Box 36, Muscat, Oman

**Keywords:** GBS, Genetic diversity, *Olea europaea*, Oman, Phylogenetics, SNPs, Wild olives, Evolution, Genetics, Plant sciences

## Abstract

The olive (*Olea europaea* L.) crop has significant cultural, nutritional, and economic importance worldwide. While its genetic diversity has been extensively studied in the Mediterranean Basin, little is known about olive germplasm in the Arabian Peninsula, including Oman, where it represents a unique environment for olive cultivation. In Oman, both wild and cultivated olives are exposed to arid conditions, which may have led to unique genetic adaptations. Leaf samples were collected from 44 olive accessions, including wild populations (MN and UT) distributed across northern and southern Oman and introduced cultivars originating from the Mediterranean Basin and cultivated in Oman, as a comparative reference. Genomic DNA was extracted and genotyping-by-sequencing (GBS) libraries were prepared and sequenced on the Illumina NovaSeq X platform. High-quality reads were aligned to the Mediterranean olive reference genome, and single-nucleotide polymorphisms (SNPs) were called, filtered for quality, and annotated to assess their genomic distribution. Genetic diversity indices, population structure (PCA, DAPC, STRUCTURE), phylogenetic relationships, analysis of molecular variance (AMOVA), pairwise Fst/PhiST, and linkage disequilibrium were calculated to evaluate variation, differentiation, and evolutionary patterns among wild and cultivated olives. After quality filtering, 167,875 high-confidence SNPs were retained. Functional annotation revealed that many variants were located in coding and regulatory regions. For linkage disequilibrium analysis, a more stringent SNP filtering scheme was applied, and genome-wide LD showed a progressive decay of r^2^ with increasing physical distance. Introduced cultivars used as comparative reference genotypes exhibited moderate genetic diversity (He = 0.100–0.162) and negative or near-zero F_IS_ values, indicating heterozygote excess likely due to clonal propagation, whereas Omani wild olive populations (MN and UT) showed markedly lower diversity (He = 0.060–0.106) and positive F_IS_ values, reflecting reduced heterozygosity and limited gene flow. Multivariate (PCA and DAPC), STRUCTURE, and phylogenetic analyses consistently revealed strong genetic differentiation between wild and cultivated olives, with AMOVA indicating that ~ 51% of genetic variation was explained by differences among predefined groups, dominated by the contrast between wild and cultivated olives. This study provides an early genome-wide SNP analysis of Omani wild and cultivated olives. Clear genetic differentiation was observed between wild and cultivated groups, with wild populations showing distinct variation. These findings can inform future conservation and breeding efforts for olive germplasm in arid environments.

**Supplementary Information:**

The online version contains supplementary material available at 10.1038/s41598-026-40849-0.

## Introduction

The olive tree (*Olea europaea* L.) is one of the oldest cultivated fruit crops, with domestication dating back over 6000 years in the Mediterranean Basin^1^. It is of substantial agricultural, nutritional, and cultural importance, producing fruit and oil prized worldwide for their health benefits and economic value. While olives are traditionally associated with the Mediterranean region, their cultivation has expanded to non-native areas, including parts of the Arabian Peninsula such as Oman, where drought-tolerant crops are vital for sustainable agriculture under arid conditions^2^^,^^3^.

Genetic variability forms the foundation for long-term progress in olive cultivation^4^. It broadens the range of traits available for developing improved cultivars, whether the goal is higher productivity, enhanced oil composition, or specific horticultural characteristics^5^. A rich genetic base also enhances the crop’s ability to adapt to changing climates and challenging growing conditions, while providing natural defenses against pests and pathogens. Safeguarding this diversity is therefore not only essential for current breeding and management strategies, but also for maintaining the reservoir of traits that future generations may depend upon^6^^,^^7^.

Cultivating crops with a well-defined genetic background is preferable for ensuring consistency, reliability, and reproducibility in agricultural systems. However, cultivating crops with a restricted genetic foundation is often more vulnerable to environmental and biological stresses, a concern that is particularly acute in arid regions such as Oman. When genetic variability is limited, the scope for selecting traits that confer tolerance to heat, drought, salinity, and emerging pests becomes narrower. This can result in uniform responses across large areas, making entire plantings susceptible to the same disease outbreak or climate anomaly. In dryland agriculture, where water scarcity and high evaporative demand are persistent challenges, such genetic uniformity can reduce the long-term stability of yields. Expanding and safeguarding the genetic base through the introduction of diverse varieties, the use of wild relatives, and targeted breeding is therefore essential to develop olive varieties that enhance resilience and ensure sustainable production under harsh growing conditions^8^^,^^9^.

Molecular markers have become indispensable for characterizing genetic diversity, population structure, and evolutionary relationships in perennial crops such as olives^10^. Unlike morphological or agronomic traits, which are often influenced by environmental conditions and cultivation practices, molecular markers provide stable and heritable indicators of genetic variation^5^. Early studies relied heavily on Random Amplified Polymorphic DNA (RAPD), Amplified Fragment Length Polymorphism (AFLP), and Simple Sequence Repeat (SSR) markers, which provided considerable insights into olive genetic diversity and domestication patterns^11,12^. However, these markers cover limited portions of the genome and often lack the resolution needed for fine-scale diversity studies. On the other hand, single-nucleotide polymorphisms (SNPs) have emerged as the preferred marker type due to their high abundance across genomes, codominant inheritance, reproducibility, and amenability to high-throughput genotyping. Advances in next-generation sequencing technologies, particularly Genotyping-by-Sequencing (GBS) and Restriction-site Associated DNA sequencing (RAD-seq), have enabled the discovery and application of thousands of SNPs across diverse olive germplasm collections^13^^,^^14^.

Recent applications of SNP-based tools in olives have demonstrated their effectiveness in resolving cultivar identities, detecting redundancy in germplasm banks, and tracing domestication histories^15^. Comparative studies also show that SNPs outperform SSRs in capturing fine-scale diversity patterns, making them particularly valuable for managing germplasm resources and designing breeding strategies tailored to local environments^10^. Thus, the integration of SNP-based molecular tools represents a transformative step in olive genetics, offering high-resolution insights necessary for conservation, cultivar improvement, and adaptation to challenging climates such as those in Oman.

The potential distinctiveness of Omani olive germplasm may be influenced by geographic isolation and the unique environmental pressures of Oman’s mountainous terrain. In wild *Olea europaea* populations of the Hajar Mountains, despite fragmentation and environmental stress, researchers detected unexpectedly high genetic variation and the absence of clonal growth, indicating a distinctive and resilient genetic structure in these isolated stands^8^.

Olive cultivation in Oman relies almost exclusively on recently introduced commercial cultivars originating from the Mediterranean Basin. These cultivars are clonally propagated genotypes and therefore do not constitute biological populations, nor do they represent locally adapted or historically established Omani germplasm. In contrast, wild olive trees occurring in Oman represent naturally reproducing units and provide a unique opportunity to investigate native genetic diversity in a marginal environment.

Accordingly, the present study focuses on assessing genome-wide genetic diversity and broad-scale genetic differentiation in Omani wild olives, while introduced cultivars are included solely as comparative reference genotypes to contextualize patterns of genetic divergence.

Despite olives being among the most important fruit crops in the Mediterranean Basin, research on olive cultivation and genetic diversity in Oman remains in its early stages. While wild *Olea europaea* subsp. *cuspidata* populations in the Hajar and Dhofar Mountains have been studied to some extent^16^, detailed investigations of cultivated Omani olive varieties remain very limited. In particular, there is a lack of genome-wide studies employing high-resolution molecular markers such as SNPs generated through genotyping-by-sequencing (GBS). Without such data, it is challenging to evaluate the genetic diversity, relatedness, and potential uniqueness of Omani olives in comparison to other Mediterranean and Asian germplasm.

Understanding the genetic diversity of Omani olives is particularly important because the country’s distinctive geography and harsh arid conditions may have led to unique adaptations in its germplasm. Characterizing these patterns is essential for identifying potentially valuable genetic resources, providing a foundation for future breeding strategies, and supporting the long-term sustainability of olive cultivation in Oman. To achieve this, the present study aimed to apply genotyping-by-sequencing (GBS) to generate SNP datasets for Omani wild olive populations, with introduced cultivars included as comparative references. This approach represents a timely and necessary step toward characterizing native genetic diversity, clarifying genetic relationships with Mediterranean and Asian germplasm, identifying potentially unique genetic resources, and informing conservation strategies as well as future, targeted crop improvement programs tailored to the harsh environmental conditions of Oman.

## Materials and methods

### Plant material, collection sites, and ethical considerations

Olive leaf samples from cultivated cultivars were collected from olive orchards on private farms, while wild olives were sampled from two regions: Utem from the Al Jabal Al Akhdar mountains (northern Oman) and Metan from the Dhofar Mountains (southern Oman) (Table 1). Both were sampled in October 2024. The cultivated samples represent recently introduced commercial olive cultivars and were included solely as a comparative reference group, as these cultivars are clonally propagated genotypes and do not constitute biological populations nor locally adapted or historically established Omani germplasm. These cultivated samples belong to *Olea europaea* subsp. *europaea* and originate from different regions of the Mediterranean Basin (e.g. Spain, Greece, and Italy). The plant material used in this study (cultivated and wild olives) is neither endangered nor protected under the IUCN Red List or CITES. The collection of olive leaves complied with institutional, national, and international guidelines. No specific permits were required for the collection of this species, as it is widely cultivated and not subject to restrictions. Wild olive leaf samples from Oman were previously collected and deposited as herbarium voucher specimens at the University of Reading Herbarium (RNG), United Kingdom^16^. Plant identity was confirmed using these reference specimens, standard taxonomic keys, and biogeographic considerations. The wild olive samples correspond to *Olea europaea* subsp. *cuspidata*. Details of the wild olive locations in Oman where samples were collected have been reported by Al-Jabri et al.^17^.

**Table 1:** Sampling information of wild and cultivated olive (*Olea europaea* subsp. *cuspidata* and subsp. *europaea*) included in this study.

Cultivar	Cultivar Code	Country of Origin	Sample	Location
Arbequina	AR	Spain	4	Al Jabal Al Akhdar Farms
Bedh AL Hamam	BH	Oman	5	Al Jabal Al Akhdar Farms
Coratina	CO	Italy	5	Al Jabal Al Akhdar Farms
Dolce	DS	Italy	5	Al Jabal Al Akhdar Farms
Koroneiki	KL	Greece	5	Al Jabal Al Akhdar Farms
Metan	MN	Oman	5	Wild, Dhofar Mountains
Picual	PC	Spain	5	Al Jabal Al Akhdar Farms
Tufahi	TF	Oman	5	Al Jabal Al Akhdar Farms
Utem	UT	Oman	5	Wild, Al Jabal Al Akhdar

The climate of Al Jabal Al Akhdar, where olive is currently cultivated, is semiarid and cool due to its elevation (> 2000 m). Winter temperatures often drop below 0 °C, providing sufficient chill units for temperate and deciduous fruit crops^18^. Olive has been recently introduced to this region as a commercial crop, with different cultivars under evaluation for oil production. Farms are typically smallholdings (< 1 acre) and located in the same areas as pomegranate orchards^19^. Olive farms follow the same traditional practices of growing other fruit crops in Al Jabal Al Akhdar^18^.

### Genomic DNA extraction and quality control

Ten milligrams (10 mg) of young leaf tissue (~ 0.5 cm × 0.5 cm) were harvested and homogenized in liquid nitrogen via bead beating. Genomic DNA was extracted using the Aprep Total DNA Kit (APBIO, Korea) for subsequent GBS library preparation. DNA concentration and integrity were evaluated using a Qubit fluorometric assay (Thermo Fisher Scientific) and agarose gel electrophoresis (1% agarose).

### Construction of GBS library and NGS

Genomic DNA was digested overnight at 37 °C using the restriction enzymes *PstI* and *MspI*. In a 96-well plate, adapters were ligated to the DNA using a reaction mixture containing T4 DNA ligase. Following ligation, the enzymes were heat-inactivated at 75 °C before proceeding to the subsequent steps. Samples were pooled and purified using HiAccuBead magnetic beads (AccuGene, Korea). The purified DNA was subsequently amplified by PCR using the following thermal cycling protocol: an initial denaturation at 95 °C for 2 min; 16 cycles of 95 °C for 30 s, 62 °C for 30 s, and 72 °C for 30 s; and a final extension at 72 °C for 5 min. The prepared GBS libraries were sequenced on the Illumina NovaSeq X platform (Illumina Inc., San Diego, CA, USA).

### Preprocessing of sequence reads

Adapter sequences were removed from the raw reads using Cutadapt v1.8.3^20^. This was followed by quality trimming and demultiplexing using Trimmomatic v0.39^21^, applying the following parameters: a sliding window of 4 bases with an average quality threshold of ≥ 15, trimming of low-quality bases from both the 5′ and 3′ ends (LEADING and TRAILING ≥ 3), and discarding of sequences shorter than 36 bp. Barcoded reads were then demultiplexed per individual sample and forwarded to downstream analytical steps.

### Alignment to the reference genome

High-quality reads that remained after preprocessing were aligned to the “Mediterranean olive” reference genome *(Olea europaea* subsp. europaea; NCBI accession GCA_902713445.1), which comprises 23 chromosomes and 9751 scaffolds, with a total length of 1,316,676,700 bp (~ 1.32 Gb)^22^. Alignment was performed with BWA-MEM2^23^^,^ and the resulting files were produced in BAM format using the software’s default configuration.

### SNP detection

To identify raw SNPs, aligned reads in BAM format were input into DeepVariant^24^, a deep learning–driven variant calling tool that interprets alignments as image-like data and applies a convolutional neural network for prediction. Following this, SNPs were validated using a custom script developed in-house by SEEDERS^25^, and subsequently extracted for downstream analysis^26^.

### SNP annotation and genomic distribution

Single nucleotide polymorphisms (SNPs) were functionally annotated using SnpEff v5.4, employing a custom-built database based on the *Olea europaea* OLEA9 genome assembly. Gene models were provided in GFF3 format and combined with the corresponding genomic FASTA sequences to generate a species-specific annotation database. Annotated variants were categorized according to their genomic locations, including intergenic, intronic, and exonic regions. The distribution of SNPs across these categories was subsequently quantified to assess their genomic context and potential functional relevance.

### Generate SNP matrix

A comprehensive SNP matrix was assembled to facilitate comparative analysis across all samples. Raw variant positions, derived by aligning each sample to the reference genome, were integrated into a unified set of candidate SNP loci. A comparative evaluation across samples was then performed to eliminate potential false-positive calls, yielding a final matrix of high-confidence SNPs. These variants were subsequently categorized based on established classification criteria.

### SNP filtering

To enhance the precision and reliability of SNP identification, the initial SNP matrix underwent rigorous filtering following the strategy previously used by Taniguti et al.^27^. Only biallelic SNP loci were retained for analysis. Further criteria included a minimum sequencing depth of 3 reads per locus, a minor allele frequency (MAF) greater than 5% to exclude rare variants, and exclusion of loci with more than 30% missing data across samples. This filtering strategy was applied to generate the primary SNP dataset used for population genetic analyses. A more stringent filtering scheme was subsequently applied specifically for linkage disequilibrium analyses to minimize genotyping noise associated with GBS data.

### Linkage disequilibrium (LD) analysis

Prior to linkage disequilibrium (LD) estimation, SNPs were stringently filtered to minimize genotyping noise inherent to GBS data. Only loci with a minimum read depth ≥ 5, minor allele frequency (MAF) ≥ 0.05, and missing data ≤ 20% were retained. LD was estimated using PLINK v1.9 by calculating pairwise r2 values between SNPs within sliding windows of 100 kb (–r2 –ld-window-kb 100). LD decay was visualized by plotting r2 values against the physical distance between SNPs, applying LOESS smoothing in R to illustrate genome-wide trends. This approach ensures that LD estimates are based on high-confidence variants and reduces the likelihood of spurious associations arising from low-depth or poorly genotyped loci.

### Advanced bioinformatics analysis

#### Genetic diversity

Genetic diversity indices were derived from the final set of filtered SNP loci to evaluate the level of variation among wild olive groups and among cultivated reference genotypes (treated as comparative units rather than biological populations). The populations module of the Stacks^28^, a core tool for population-level genetic analysis, was used to compute key diversity metrics. These included: observed heterozygosity (Ho), expected heterozygosity (He), nucleotide diversity (π), and the inbreeding coefficient (F_IS_). These indices provide essential insights into genetic variation, mating patterns, and the evolutionary potential of the studied olive populations.

#### Population genetic structure

Bayesian inference of genetic structure was conducted using fastSTRUCTURE^29^, as a complementary exploratory analysis. Given the clonal propagation of cultivated olive varieties and the relatedness among individuals belonging to the same genotype, STRUCTURE results were interpreted cautiously. Analyses focused on identifying broad-scale genetic differentiation, and the most biologically meaningful solution was retained based on model support. The optimal number of clusters (K) was determined using the choose.py script. STRUCTURE outputs were visualized using StructureSelector^30^. The most biologically relevant clustering solution (K = 3) is presented in the main text, with additional clustering results provided in the Supplementary Materials.

#### Principal component analysis (PCA)

Principal Component Analysis (PCA) and Discriminant Analysis of Principal Components (DAPC) were conducted using the adegenet package in R^31^. PCA was employed to reduce the dimensionality of the SNP dataset and to summarize the overall patterns of genetic variation among samples. Subsequently, DAPC was applied to identify and describe distinct genetic clusters by maximizing between-group variation while minimizing within-group variation. These analyses provided robust insights into population structure and genetic differentiation across the studied olive populations. In this study, PCA was used as the primary ordination method, while DAPC results were interpreted cautiously and used to support patterns observed in PCA.

#### Construction of phylogenetic tree

Phylogenetic relationships among the sampled individuals were inferred using the final set of filtered SNP loci. An Unweighted Pair Group Method with Arithmetic Mean (UPGMA) tree was constructed using the R package poppr^32^, incorporating 999 bootstrap replicates to assess branch support. Additionally, a Maximum Likelihood (ML) phylogenetic tree was generated using IQ-TREE2^33^ with 1000 bootstrap replicates. The GTR + ASC substitution model was applied, which is specifically appropriate for SNP datasets due to its ability to correct for ascertainment bias. Non-informative SNP positions were automatically excluded during the analysis to improve resolution and clarity. Both phylogenetic trees were visualized as unrooted circular trees using the ape package in R^34^, allowing for an intuitive and comprehensive representation of the genetic relationships among individuals. Phylogenetic clustering was interpreted in terms of genetic similarity among individuals rather than population-level evolutionary relationships.

#### Analysis of Molecular Variance (AMOVA)

To assess the extent of genetic differentiation among individuals grouped by environmental or biological classifications, an Analysis of Molecular Variance (AMOVA) was performed using the final set of filtered SNP loci. AMOVA was conducted to partition genetic variance among predefined biological groups, primarily contrasting wild olives and cultivated reference genotypes. The analysis was conducted using the poppr package in R^32^, which partitions genetic variance at multiple hierarchical levels, including among groups, within groups, and within individuals.

#### Pairwise Fst and PhiST

Genetic differentiation between population pairs was evaluated using the final filtered set of SNP markers in conjunction with population metadata. This analysis was conducted using the Populations module in the Stacks^28^, which calculates pairwise Fst and PhiST values to assess the degree of genetic divergence among the studied populations. Fst measures the proportion of genetic variance that can be attributed to population differences, while PhiST incorporates both genetic distance and evolutionary divergence among alleles. Pairwise Fst and PhiST values were interpreted as measures of genetic differentiation among predefined sample groups rather than true evolutionary populations.

## Results

### High alignment efficiency to the olive reference genome

The average of each sample yielded approximately 4.97 million high-quality trimmed reads, of which 96.04% successfully aligned to the reference genome of *Olea europaea* subsp. *europaea* (Mediterranean olive), resulting in an average of 4.82 million mapped reads per sample (Table 2). Overall, 218.5 million reads were retained after trimming across all samples, with 212.1 million (∼97%) successfully mapped to the genome, indicating good sequence quality and compatibility of the samples with the reference genome.

**Table 2:** Summary of alignment to the reference genome.

	No. of trimmed reads	No. of mapped reads	Percentage of mapped reads
Average	4,965,906	4,821,196	96.04%
Sum	218,499,880	212,132,611	–

### Stringent filtering yielded a high-quality SNP dataset

A total of 2,977,216 single-nucleotide polymorphisms (SNPs) were initially identified from the raw dataset. To ensure data quality and remove low-informative markers, a series of filtering steps was applied. Filtering thresholds were chosen to minimize the inclusion of rare variants and missing genotypes, which may arise from sequencing errors or low coverage. Specifically, SNPs with minor allele frequency (MAF) below 5%, missing data greater than 30% across individuals, or read depth less than 3 were excluded. Filtering was performed using VCFtools vX.X and custom Python scripts to ensure reproducibility.

After applying these criteria, 1,914,463 SNPs remained following the MAF filter, and 234,162 SNPs passed the missing data filter. Finally, applying all filters simultaneously yielded a high-quality dataset of 167,875 SNP loci, which was subsequently used for downstream population genetic analyses (Table 3).

**Table 3:** Summary of SNP filtering process in 44 olive samples.

Step	Filtering criteria	Number of SNP loci
1	Total SNP matrix	2,977,216
2	MAF (minor allele frequency) > 5%	1,914,463
3	Missing data < 30%	234,162
4	MAF > 5% and Missing data < 30%	167,875

### Functional annotation of SNPs reveals genomic distribution and potential effects

A total of 167,875 high-quality SNPs were annotated using SnpEff. Annotated variants were classified according to their genomic locations, including exons, introns, untranslated regions (UTRs), upstream and downstream regulatory regions, splice sites, intergenic regions, and non-coding transcripts (Table 4). Overall, more than one-third of the annotated SNPs were located within gene regions, with the majority of genic variants occurring in exonic regions, followed by intronic and untranslated regions. Exonic SNPs included both synonymous and non-synonymous substitutions, reflecting potential functional impacts on coding sequences. The presence of variants in both regulatory and coding regions indicates that the dataset captures biologically relevant genomic variation.

**Table 4:** Distribution of SNPs across genomic regions as determined by SnpEff.

Genomic Region	Count	Percent (%)
Exon	40,251	28.77
Intron	8270	5.91
5′ UTR	7618	0.54
3′ UTR	13,643	0.97
Upstream	29,316	20.95
Downstream	37,332	26.68
Intergenic	6723	4.81
Splice site	3229	0.23
Transcript (non-coding/ intragenic)	15,557	11.12

### Cultivated olive reference genotypes exhibit higher genetic diversity than wild populations

Expected (He) and observed heterozygosity (Ho), fixation index (F_IS_), and nucleotide diversity (π) were calculated based on filtered 167,875 SNPs for each predefined sample group (Table 5).

**Table 5:** Genetic diversity indices of olive populations.

Population	Expected heterozygosity (He)	Observed heterozygosity (Ho)	Inbreeding coefficient (F_IS_)	Nucleotide diversity (π)
AR	0.100	0.135	– 0.026	0.100
BH	0.139	0.172	– 0.015	0.139
CO	0.138	0.149	0.022	0.138
DS	0.162	0.148	0.079	0.162
KL	0.130	0.207	– 0.109	0.130
MN	0.060	0.056	0.031	0.060
PC	0.144	0.169	0.004	0.144
TF	0.084	0.128	– 0.058	0.084
UT	0.106	0.091	0.060	0.106
Average	0.118	0.140	– 0.001	0.118

From a total of 167,875 SNPs identified in *Olea europaea* samples, the mean observed Ho was 0.140, ranging from 0.056 to 0.207. The mean He was 0.118, with values ranging from 0.060 to 0.162. The average F_IS_ was close to zero, ranging from –0.109 to 0.060. Across the nine predefined sample groups analyzed, the mean of nucleotide diversity (π) was 0.118, with a range of 0.060–0.162.

The cultivated olive reference genotypes generally exhibited moderate levels of genetic diversity. Expected heterozygosity (He) ranged from 0.100 (AR) to 0.162 (DS), while observed heterozygosity (Ho) was equal to or slightly higher than He in most cultivated genotypes. Given that these samples represent clonally propagated cultivars rather than true biological populations, diversity indices and F_IS_ values are interpreted descriptively and reflect genotype-level variation rather than population-level evolutionary processes.

In contrast, the two wild populations (MN and UT) exhibited lower genetic diversity across all measured indices. MN showed the lowest values (He = 0.060, Ho = 0.056, π = 0.060), whereas UT displayed slightly higher diversity (He = 0.106, Ho = 0.091, π = 0.106), but still lower than most cultivated genotypes. Both wild populations had positive F_IS_ values (MN = 0.031; UT = 0.060), indicating a mild deficit of heterozygotes, likely due to limited gene flow or small effective population sizes associated with geographic isolation.

Overall, these results highlight the reduced genetic diversity of wild olive populations compared to cultivated reference genotypes. However, diversity metrics for cultivated samples should be interpreted cautiously, as they reflect clonal, genotype-level variation rather than population-level evolutionary processes.

### Population structure reveals a clear separation between wild and cultivated olives

Analysis of 167,875 SNPs in 44 individuals was conducted using fastSTRUCTURE to provide an overview of genetic relationships among wild and cultivated olives. Based on trends in marginal likelihood and cross-validation error (Supplementary Figure S1), K = 3 was considered the most informative clustering solution.

**Fig. 1:**
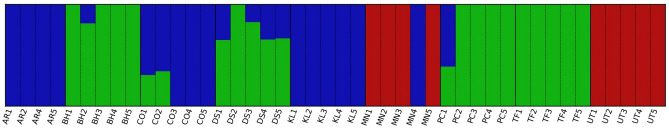
Population genetic structure at K = 3. Population genetic structure of 44 olive individuals inferred using fastSTRUCTURE at K = 3. Each vertical bar represents an individual, and colors indicate the proportion of ancestry assigned to each genetic cluster.

At K = 3, wild olive populations (MN and UT) formed a distinct genetic cluster clearly separated from cultivated reference genotypes (AR, BH, CO, DS, KL, PC, TF) (Fig. 1). Limited admixture was observed in some cultivated accessions, while MN showed minor mixed ancestry.

Population structure patterns across a wider range of K values (K = 2–10) are shown in Supplementary Figure S2 and reveal additional substructure among cultivated genotypes, while maintaining the clear differentiation between wild and cultivated olives.

### Genome-wide linkage disequilibrium decay in Omani olives

**Fig. 2:**
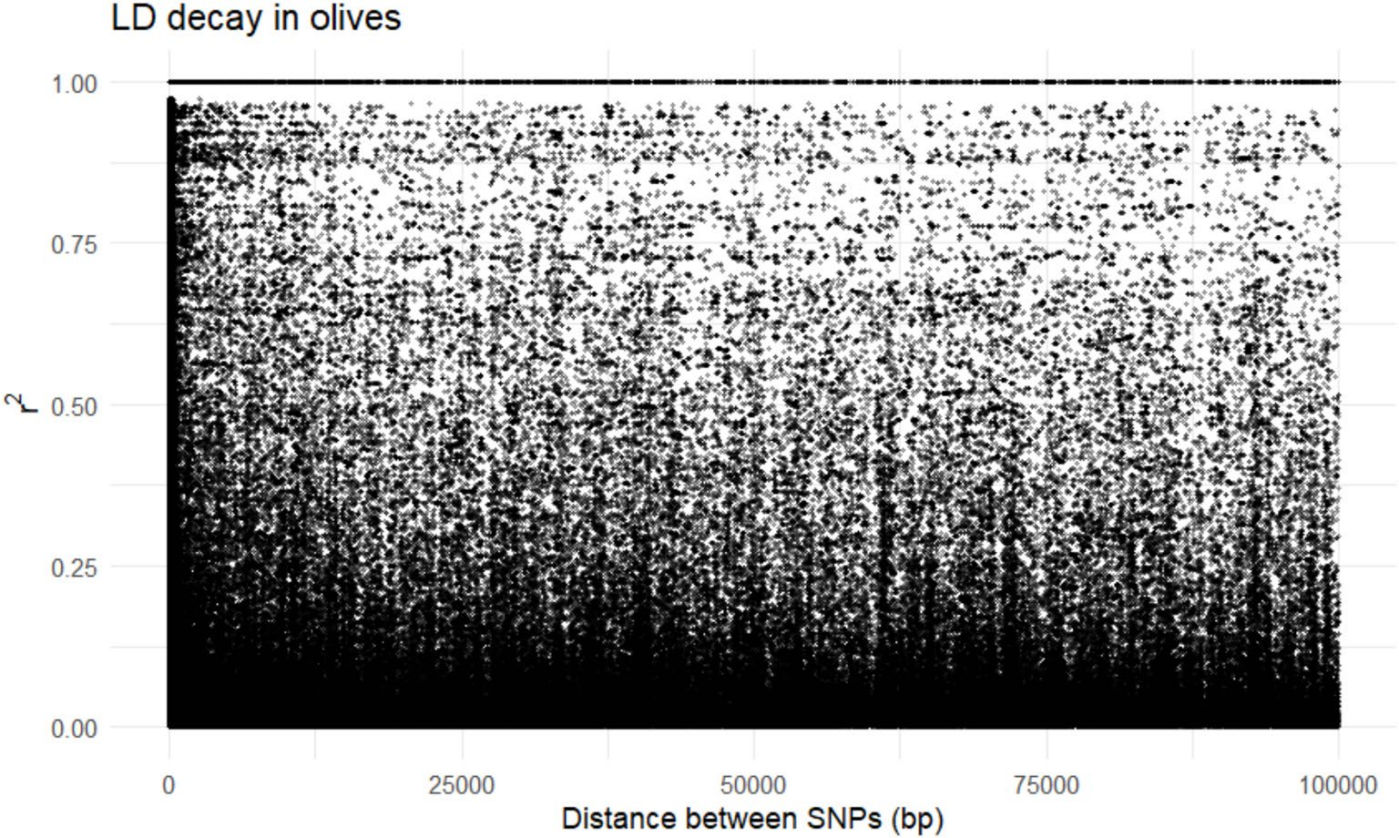
Genome-wide linkage disequilibrium (LD) decay in Omani olives. Pairwise r^2^ values are plotted against physical distance (bp) between SNPs up to 100 kb. Each point represents a SNP pair, and the overall trend illustrates a gradual decay of LD with increasing physical distance, consistent with expectations for genome-wide SNP data generated using GBS.

A total of 53,106 high-quality SNPs passed the final filtering criteria and were used for LD analysis. The LD decay plot (Fig. 2) revealed a clear relationship between pairwise r^2^ values and physical distance between SNPs. Higher LD values were predominantly observed at short physical distances, followed by a gradual decline as the distance between SNPs increased, reaching lower r^2^ values at larger distances (up to 100 kb).

This pattern indicates a progressive breakdown of linkage disequilibrium with increasing physical distance, consistent with expectations for genome-wide SNP datasets generated using GBS approaches. The observed LD decay suggests that the retained marker set captures meaningful genome-wide linkage relationships and is not dominated by noise arising from low-depth or poorly genotyped loci.

### Principal component analysis highlights divergence of Omani wild olives from introduced cultivars

**Fig. 3:**
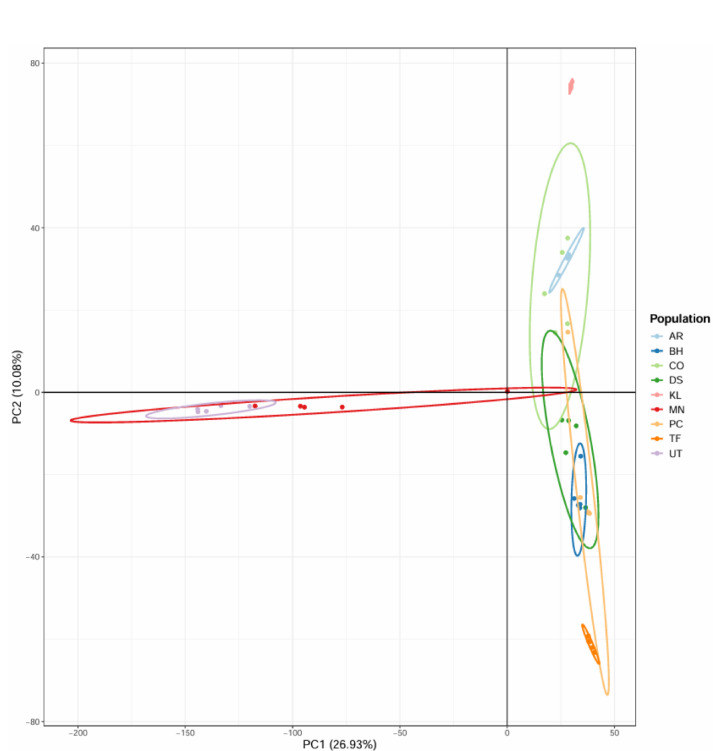
Principal component analysis (PCA) of 44 olive samples using filtered SNPs across PC1 and PC2.

Figure 3 shows the principal component analysis (PCA) of 44 olive samples using 167,875 filtered SNP loci. The first two principal components explained 26.93% (PC1) and 10.08% (PC2) of the total genetic variability, respectively. Each point represents an individual, while ellipses indicate 95% confidence intervals for each population.

Consistent with the study’s focus, the PCA reveals clear genetic divergence between the Omani wild olive populations (MN and UT) and the introduced cultivars, which are included solely as comparative reference genotypes. The UT wild population is the most genetically distinct, positioned farthest from the cultivated cluster on PC1, reflecting its unique genetic background. The MN population is closer to the cultivated varieties, with the exception of individual MN4, which clusters within the cultivated group, potentially indicating admixture or mislabeling.

The introduced cultivars (AR, BH, CO, DS, KL, PC and TF) form a tight cluster on the right side of the plot, consistent with their clonal propagation and recent introduction from Mediterranean regions. This clustering reflects low within-group variability among the cultivars, as expected given their origin and propagation method.

### DAPC confirms genetic divergence of Omani wild olives and similarity of introduced cultivars

Discriminant analysis of principal components (DAPC) was conducted on the 44 olive samples. The first discriminant function (LD1) explained most of the between-group variability, with approximately 40 PCs retained to capture nearly 100% of the total genetic variance.

**Fig. 4:**
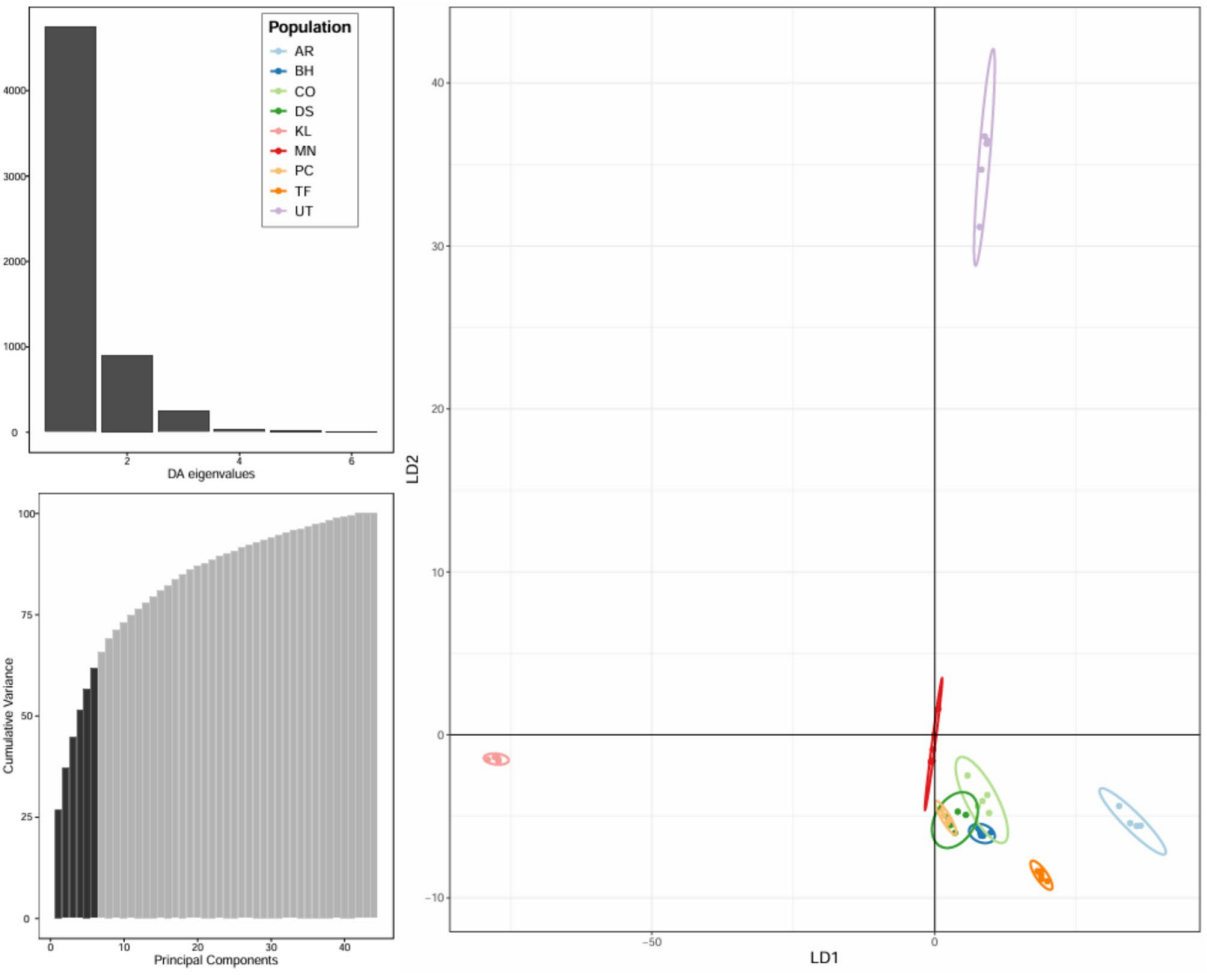
Discriminant analysis of principal components (DAPC) for 44 olive samples using filtered SNPs.

The DAPC scatterplot (LD1 vs. LD2) shows clear separation between the Omani wild olives and the introduced cultivars. The UT population is well separated along LD2, consistent with its distinct wild gene pool. The MN population shows partial separation, with individual MN4 clustering near the introduced cultivars, supporting the possibility of admixture or misclassification. The introduced cultivars form a tight cluster, confirming their genetic similarity due to clonal propagation and shared Mediterranean origin (Fig. 4).

### Phylogenetic tree reveals divergence of Omani wild olives from introduced cultivars

**Fig. 5:**
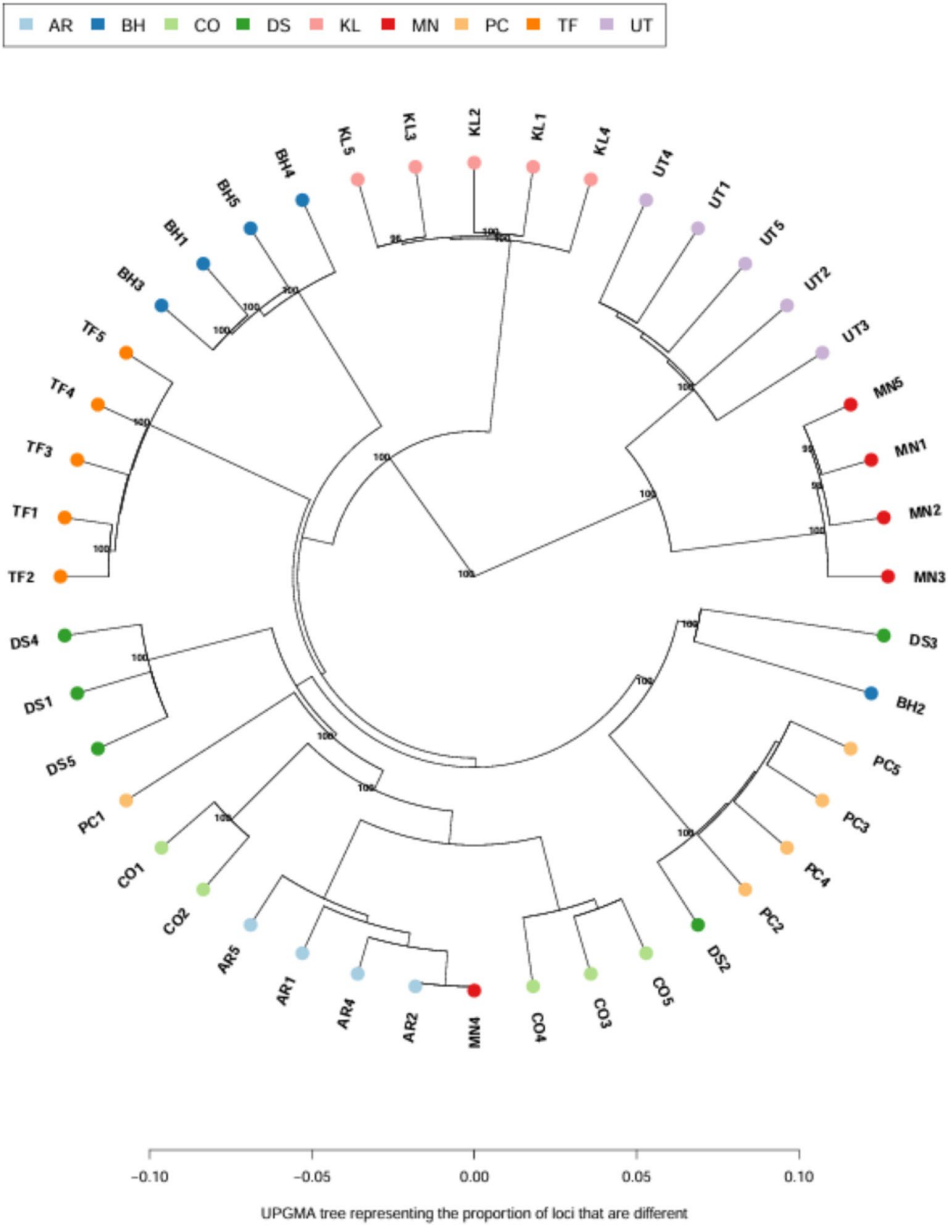
UPGMA tree analysis of 44 olive samples using filtered SNPs. Numbers on the branch nodes indicate bootstrap value percentage.

To infer genetic relationships among the 44 olive genotypes, a UPGMA tree was constructed using pairwise genetic distances based on 167,875 SNP loci (Fig. 5). The tree shows a clear separation between Omani wild olives (MN and UT) and the introduced cultivars, which were included as comparative reference genotypes. Both wild groups are positioned on long, distinct branches, indicating deep genetic divergence from the cultivated material. Among the wild olives, UT appears the most divergent, while MN is closer to the cultivated cluster, with the exception of individual MN4, which clusters with cultivated genotypes, suggesting possible admixture or mislabeling.

Introduced cultivars generally cluster together, reflecting their clonal propagation and shared Mediterranean origin. However, a few inconsistencies were observed, such as unexpected clustering of individual DS2 with Picual and the splitting of Coratina accessions into two subclusters, which may reflect technical artefacts related to SNP calling or sequencing depth rather than true biological differentiation. High bootstrap values support the major groupings observed in the tree.

### AMOVA indicates that most genetic variability is partitioned between wild olives and introduced cultivars

Analysis of Molecular Variance (AMOVA) showed that 50.72% of the total genetic variability was attributed to differences among groups, while 45.09% occurred within individuals and only 4.19% among individuals within groups (Table 6). This pattern reflects strong genetic differentiation among Omani wild olives and the introduced cultivars used as reference genotypes.

**Table 6:** AMOVA results showing genetic differentiation between Omani wild olives and introduced cultivars.

	df	Sum of Squares	Variance component	Variation (%)
Among populations	8	1,134,723	13,100.89	50.72
Among individuals within populations	35	483,313.7	1081.34	4.19
Within individuals	44	512,436.2	11,646.28	45.09
Total	87	2,130,473	25,828.51	100

The low proportion of variability among individuals within groups is consistent with the clonal nature of the cultivated material and the limited sample size of the wild populations. The PhiST value (0.507) confirms substantial genetic differentiation among groups, whereas the low PhiST value (0.085) indicates high genetic similarity among individuals within groups. These results should be interpreted with caution, as the AMOVA framework treats introduced cultivars as groups, which do not represent natural populations but rather clonally propagated reference genotypes. Nevertheless, the analysis robustly supports deep genetic divergence between Omani wild olives and cultivated olives.

### Pairwise differentiation highlights strong divergence of Omani wild olives from introduced cultivars

The levels of Fst and PhiST, estimated based on 167,875 SNPs, are presented in Table 7. These indices assess the degree of genetic differentiation among predefined groups, with higher values indicating stronger genetic divergence. Pairwise comparisons reveal consistently high differentiation between the Omani wild olives (MN and UT) and the introduced cultivars used as reference genotypes. Both MN and UT show elevated Fst and PhiST values relative to all cultivated varieties, reflecting their distinct wild genetic backgrounds. Among the wild samples, UT shows uniformly high differentiation from the cultivated group, while MN displays similarly high values, with some variation among comparisons.

**Table 7:** Pairwise differentiation between Omani wild olives and introduced cultivars.

Pop	AR	BH	CO	DS	KL	MN	PC	TF	UT
AR	–	0.296	0.254	0.222	0.313	0.613	0.262	0.426	0.568
BH	0.197	–	0.236	0.165	0.312	0.521	0.175	0.297	0.505
CO	0.162	0.152	–	0.182	0.258	0.530	0.219	0.361	0.509
DS	0.132	0.094	0.106	–	0.241	0.464	0.106	0.217	0.459
KL	0.222	0.231	0.174	0.152	–	0.580	0.282	0.424	0.554
MN	0.489	0.424	0.416	0.372	0.471	–	0.498	0.652	0.439
PC	0.166	0.113	0.138	0.053	0.195	0.409	–	0.269	0.488
TF	0.314	0.214	0.255	0.129	0.337	0.562	0.191	–	0.604
UT	0.461	0.415	0.402	0.362	0.460	0.329	0.400	0.520	–

*Below the diagonal line, the left-below values represent pairwise Fst between populations, while the right-above values indicate pairwise PhiST values.

In contrast, relatively low differentiation values are observed among several cultivated varieties (e.g., DS, PC, and BH). For example, the DS-PC comparison shows the lowest Fst value (0.053), consistent with their shared Mediterranean origin and clonal propagation rather than indicating ongoing gene flow. Overall, the pairwise differentiation matrix highlights a clear pattern of strong genetic divergence between Omani wild olives and introduced cultivars, while differences among cultivated varieties remain comparatively low and should be interpreted cautiously given their clonal nature.

## Discussion

This study presents the first comprehensive genome-wide SNP-based analysis of both wild and cultivated olive germplasm in Oman, building upon earlier SNP-based work that focused exclusively on wild populations^16^. By analyzing more than 167,000 high-quality SNPs generated through genotyping-by-sequencing (GBS), we uncover new insights into the diversity, broad-scale differentiation, and evolutionary relationships of Omani olives. Our results reveal that wild populations (MN and UT) are genetically distinct but comparatively less diverse, while introduced cultivars are included solely as comparative reference genotypes and exhibit moderate diversity, reflecting multiple introductions and some substructure. Together, these findings highlight the dual importance of conserving Oman’s unique wild germplasm and contextualizing cultivated diversity for comparative purposes, supporting breeding and agricultural sustainability.

The cultivated genotypes analyzed in this study exhibited moderate genetic diversity, which is lower than the values reported for Mediterranean germplasm^35^. Similarly, other Mediterranean collections genotyped with genome-wide SNPs revealed higher diversity indices^14^. The comparatively lower diversity in Omani introduced cultivars likely reflects recent introduction, a small germplasm base, and founder effects, in contrast to the long history of olive domestication in the Mediterranean.

Interestingly, some introduced cultivars, such as DS, exhibited relatively high expected heterozygosity (He = 0.162), indicating potential admixture, while others, such as AR and TF, showed lower He but still moderate observed heterozygosity, reflecting potential bottlenecks followed by clonal propagation. These contrasting patterns highlight the heterogeneous nature of Omani introduced cultivars, likely derived from multiple introductions and farmer selection processes.

In contrast, the two wild populations (MN and UT) displayed markedly lower diversity consistent with long-term isolation and small effective population sizes. MN shows the lowest diversity, whereas UT is slightly more diverse. Positive F_IS_ values suggest a deficit of heterozygotes, likely due to inbreeding or restricted gene flow. Overall, wild populations represent genetically distinct and vulnerable germplasm that requires conservation attention.

Nevertheless, phylogenetic trees, PCA, and STRUCTURE analyses consistently show that the MN and UT wild olives are highly divergent from the introduced cultivars, with UT generally appearing the most isolated, while MN shows clear divergence except for individual MN4, which clusters closer to the introduced cultivars. A few additional minor inconsistencies, such as DS2 grouping with Picual or the splitting of Coratina accessions into two subclusters, likely reflect methodological noise rather than true biological differentiation. This pattern is consistent with earlier studies of wild *Olea europaea* subsp. *cuspidata* in the Hajar Mountains, which reported unique genetic signatures shaped by geographic isolation^8^^,^^16^. The strong divergence of Omani wild olives underscores their evolutionary significance, as they may carry alleles related to stress tolerance -especially drought and heat- that could be valuable for breeding programs in arid regions.

The STRUCTURE analysis, while consistent with PCA, is presented here primarily for completeness. It does not provide additional insights into the differentiation between wild and introduced cultivars, which is already clearly captured by PCA. At K = 2, STRUCTURE separated wild and introduced olives, reproducing the major pattern already evident from PCA and phylogenetic analyses. This pattern is consistent with previous reports showing clear genetic separation between wild and cultivated olives in the Mediterranean and Africa^14,22^. At K = 3, limited substructure was observed among introduced cultivars, reflecting multiple introduction events and admixture, a pattern commonly reported in olive germplasm shaped by domestication history and clonal propagation^11^^,^^22^.

Higher K values (4–10) revealed fine-scale substructure within introduced cultivars, which likely represents intra-group diversity rather than distinct evolutionary lineages, as also observed in high-resolution SNP studies^9^. These higher K solutions are therefore presented for completeness only and are not interpreted biologically in this study.

Overall, the results indicate that wild Omani olives are genetically distinct and should be prioritised for conservation, whereas introduced cultivars exhibit admixture consistent with multiple introduction events. The structural patterns resemble those reported for Mediterranean germplasm, though at a comparatively lower level of diversity, reflecting recent introductions and potential founder effects.

The AMOVA results showed that most genetic variation (50.72%) occurred among populations, while 45.09% was within individuals, and only 4.19% was among individuals within populations. This partitioning reflects genetic differentiation primarily between wild Omani olives and introduced cultivars. The high PhiST (0.507) supports detectable divergence between these two groups. This pattern is consistent with recent SNP-based studies; for instance, Zunino et al.^36^ documented clear genetic separation between wild and cultivated Western Mediterranean olives, based on pairwise Fst estimates and population structure analyses.

Importantly, the LD results support the biological interpretation of the dataset, particularly in the context of clonally propagated cultivated olives. After filtering, the LD structure does not show abnormal inflation that would suggest spurious differentiation among cultivated genotypes. This indicates that the applied filtering strategy successfully reduced false signals of genetic divergence and improved the overall reliability of downstream population genetic analyses. The gradual decay of r^2^ with increasing physical distance reflects the combined effects of recombination, marker density, and SNP filtering, and is consistent with comparable LD decay patterns reported in olive when high-quality SNP datasets were used in GBS-based studies^5^. Although LD was not used directly to infer recombination rates or demographic history, this analysis provides an important quality control step and confirms that the SNP dataset is suitable for comparative analyses between wild olive populations and introduced cultivated genotypes.

Traditionally, olive diversity has been studied using SSRs, which have revealed moderate diversity but sometimes struggle to resolve fine-scale structure or discriminate closely related cultivars^37^. By contrast, SNP-based approaches offer higher resolution and reproducibility, allowing a more detailed characterization of genetic differentiation in both wild and cultivated olives. Comparative studies have consistently shown that SNPs outperform SSRs in capturing fine-scale genetic patterns and are therefore more suitable for germplasm characterization, management, and breeding design^15^^,^^38^. In this context, the successful application of GBS in the present study highlights its utility as a cost-effective and informative approach for olive genomics, especially in under-studied regions such as Oman.

The functional annotation of SNPs supports the quality of the dataset, with a substantial proportion located within gene regions. Many of these genic SNPs occur in exonic sequences, including both synonymous and non-synonymous substitutions, indicating that the marker set captures variation in potentially functional regions of the genome, as reported in other olive SNP studies^39^, although variation in proportions has been observed depending on the reference genome, marker density, and filtering strategy applied. Although the present study did not aim to directly link specific variants to phenotypic traits, the presence of numerous coding-region SNPs suggests that the dataset may be informative for future functional and association-based analyses.

The findings of this study have important implications for conservation and breeding strategies in arid environments. From a conservation perspective, wild olive germplasm represents a valuable reservoir of genetic variation, consistent with broader evidence showing that wild relatives often harbor alleles associated with adaptation to stressful conditions^7^. In this context, conservation strategies in Oman should emphasize the protection of wild olive stands in the Hajar Mountains through in situ measures, complemented by ex situ conservation efforts aimed at safeguarding genetic resources against erosion.

From a breeding perspective, the genetic contrast between wild olives and introduced cultivars highlights opportunities for exploiting wild germplasm as a source of adaptive traits. Given Oman’s arid climate, characteristics such as drought tolerance, heat resistance, and water-use efficiency are of particular relevance. Previous physiological and genomic studies suggest that wild olives persisting in extreme environments may possess alleles contributing to such adaptations^40^. Although further functional validation is required, the careful integration of wild genetic resources into breeding programs could support the development of olive cultivars better suited to local climatic conditions, in line with national strategies for agricultural diversification.

While this study provides valuable insights, several limitations should be acknowledged. First, the sample size (44 individuals, including wild trees and introduced cultivars as reference genotypes) may not capture the full extent of diversity. The limited number of individuals in both wild and introduced groups reduces statistical power for population-genomic inferences; however, this restriction reflects the need to conserve natural populations and preserve genetic diversity in the wild. Nonetheless, the dataset provides useful initial insights into patterns of differentiation and diversity in Omani olives. Second, relying solely on SNP data does not account for phenotypic traits or adaptive performance under field conditions. Third, although GBS is powerful, it represents a reduced genomic representation; whole-genome resequencing would enable more profound exploration of diversity and selection.

Future research should expand the sampling of both wild olives and introduced cultivars across Oman, integrate genomic data with phenotypic and environmental information, and apply genome-wide association studies (GWAS) to identify loci associated with agronomic traits^41^. Comparative analyses with Mediterranean and Asian germplasm will further clarify the origins and uniqueness of Omani olives.

Overall, this study provides a genomic baseline for understanding olive genetic diversity in Oman and highlights the importance of conserving wild germplasm while contextualizing introduced cultivars for comparative purposes.

## Supplementary Information

Supplementary Information.

## Data Availability

The raw sequencing data generated in this study have been deposited in the NCBI Sequence Read Archive (SRA) under the BioProject accession number PRJNA1346685. Each sample is available under its corresponding BioSample accession number. The data will be made publicly available upon publication of this article, in accordance with the NCBI release policy. Processed SNP data and related analyses are available from the corresponding author upon reasonable request.

## References

[1] Kostelenos, G. & Kiritsakis, A. Olive tree history and evolution. In *Olives and olive oil as functional foods: bioactivity, chemistry and processing* 1–12 (Wiley, 2017).

[2] Martins, S., Pereira, S., Dinis, L.-T. & Brito, C. Enhancing olive cultivation resilience: Sustainable long-term and short-term adaptation strategies to alleviate climate change impacts. *Horticulturae* **10**(10), 1066 (2024).

[3] Uylaşer, V. & Yildiz, G. The historical development and nutritional importance of olive and olive oil constituted an important part of the Mediterranean diet. *Crit. Rev. Food Sci. Nutr.* **54**(8), 1092–1101 (2014). 24499124 10.1080/10408398.2011.626874

[4] Ben Ayed, R., Ercişli, S., Hanana, M., Rebai, A. & Moreau, F. Assessment of population structure, genetic diversity and relationship of Mediterranean olive accessions using SSR markers and computational tools. *Biotechnol. Lett.* **44**(1), 113–127 (2022). 34761348 10.1007/s10529-021-03204-z

[5] Zhu, S., Niu, E., Shi, A. & Mou, B. Genetic diversity analysis of olive germplasm (*Olea europaea* L.) with genotyping-by-sequencing technology. *Front. Genet.* **10**, 755 (2019). 31497033 10.3389/fgene.2019.00755 PMC6712157

[6] Besnard, G., Terral, J.-F. & Cornille, A. On the origins and domestication of the olive: A review and perspectives. *Ann. Bot.* **121**(3), 385–403 (2018). 29293871 10.1093/aob/mcx145 PMC5838823

[7] Tadić, J. et al. Comparative analysis of cultivated and wild olive genotypes to salinity and drought stress. *Front. Plant Sci.* **15**, 1423761 (2024). 39081524 10.3389/fpls.2024.1423761 PMC11286399

[8] Habib, N. A. et al. Genetic diversity and differentiation of *Olea europaea* subsp. cuspidata (Wall. & G. Don) Cif. in the Hajar Mountains of Oman. *Genet. Resour. Crop Evol.* **68**(3), 865–883 (2021).

[9] Islam, A. F., Sanders, D., Mishra, A. K. & Joshi, V. Genetic diversity and population structure analysis of the USDA olive germplasm using genotyping-by-sequencing (GBS). *Genes* **12**(12), 2007 (2021). 34946959 10.3390/genes12122007 PMC8701156

[10] Sion, S. et al. How to choose a good marker to analyze the olive germplasm (*Olea europaea* L.) and derived products. *Genes* **12**(10), 1474 (2021). 34680869 10.3390/genes12101474 PMC8535536

[11] Baldoni, L. et al. Genetic structure of wild and cultivated olives in the central Mediterranean basin. *Ann. Bot.* **98**(5), 935–942 (2006). 16935868 10.1093/aob/mcl178 PMC2803593

[12] Fernández i Martí, A., Font i Forcada, C., Socias i Company, R. & Rubio-Cabetas, M. J. Genetic relationships and population structure of local olive tree accessions from Northeastern Spain revealed by SSR markers. *Acta Physiol. Plant.* **37**(1), 1726 (2015).

[13] Friel, J., Bombarely, A., Fornell, C. D., Luque, F. & Fernández-Ocaña, A. M. Comparative analysis of genotyping by sequencing and whole-genome sequencing methods in diversity studies of *Olea europaea* L. *Plants* **10**(11), 2514 (2021). 34834877 10.3390/plants10112514 PMC8622120

[14] El Bakkali, A. et al. Characterization of Worldwide Olive Germplasm Banks of Marrakech (Morocco) and Córdoba (Spain): Towards management and use of olive germplasm in breeding programs. *PLoS ONE* **14**(10), e0223716 (2019). 31622375 10.1371/journal.pone.0223716 PMC6797134

[15] Belaj, A. et al. Utility of EST-SNP markers for improving management and use of olive genetic resources: A case study at the Worldwide Olive Germplasm Bank of Córdoba. *Plants* **11**(7), 921 (2022). 35406901 10.3390/plants11070921 PMC9002360

[16] Al Jabri, T. A. H. *Morphological and genetic diversity of wild olive (Olea europaea subsp. cuspidata (Wall. & G. Don) Cif. in Oman.* University of Reading (2024).

[17] Al-Jabri, T., Culham, A. & Ellis, R. Wild olive in Oman and its conservation a review. *J. Agric. Mar. Sci.* **29**(1), 1–14 (2024).

[18] Al Said, F., Al-Yahyai, R. and Opara, U. 'Traditional cultivation of pomegranate in Oman’. *II All Africa Horticulture Congress 1007*, 549–555 (2012).

[19] Al-Yahyai, R., Al-Said, F. & Opara, L. Fruit growth characteristics of four pomegranate cultivars from northern Oman. *Fruits* **64**(6), 335–341 (2009).

[20] Martin, M. Cutadapt removes adapter sequences from high-throughput sequencing reads. *EMBnet. journal* **17**(1), 10–12 (2011).

[21] Bolger, A. M., Lohse, M. & Usadel, B. Trimmomatic: A flexible trimmer for Illumina sequence data. *Bioinformatics* **30**(15), 2114–2120 (2014). 24695404 10.1093/bioinformatics/btu170 PMC4103590

[22] Julca, I. et al. Genomic evidence for recurrent genetic admixture during the domestication of Mediterranean olive trees (*Olea europaea* L.). *BMC Biol.* **18**(1), 148 (2020). 33100219 10.1186/s12915-020-00881-6 PMC7586694

[23] Vasimuddin, M., Misra, S., Li, H. & Aluru, S. Efficient architecture-aware acceleration of BWA-MEM for multicore systems. In *2019 IEEE international parallel and distributed processing symposium (IPDPS)*, 314–324 (IEEE, 2019).

[24] Yun, T. et al. Accurate, scalable cohort variant calls using DeepVariant and GLnexus. *Bioinformatics* **36**(24), 5582–5589 (2020). 10.1093/bioinformatics/btaa1081 PMC8023681 33399819

[25] Kim, J.-E., Oh, S.-K., Lee, J.-H., Lee, B.-M. & Jo, S.-H. Genome-wide SNP calling using next generation sequencing data in tomato. *Molecules Cells* **37**(1), 36–42 (2014). 24552708 10.14348/molcells.2014.2241 PMC3907006

[26] Oh, J.-E. et al. Development of an SNP marker set for marker-assisted backcrossing using genotyping-by-sequencing in tetraploid perilla. *Mol. Genet. Genomics* **298**(6), 1435–1447 (2023). 37725237 10.1007/s00438-023-02066-6

[27] Taniguti, C. H. et al. Developing best practices for genotyping-by-sequencing analysis using linkage maps as benchmarks, *bioRxiv*, pp. 2022.11.24.517847 (2022). 10.1093/gigascience/giad092 PMC10603770 37889010

[28] Rochette, N. C., Rivera‐Colón, A. G. & Catchen, J. M. Stacks 2: Analytical methods for paired‐end sequencing improve RADseq‐based population genomics. *Mol. Ecol.* **28**(21), 4737–4754 (2019). 31550391 10.1111/mec.15253

[29] Raj, A., Stephens, M. & Pritchard, J. K. FastSTRUCTURE: Variational inference of population structure in large SNP data sets. *Genetics* **197**(2), 573–589 (2014). 24700103 10.1534/genetics.114.164350 PMC4063916

[30] Puechmaille, S. J. The program structure does not reliably recover the correct population structure when sampling is uneven: Subsampling and new estimators alleviate the problem. *Mol. Ecol. Resour.* **16**(3), 608–627 (2016). 26856252 10.1111/1755-0998.12512

[31] Jombart, T. & Ahmed, I. adegenet 1.3-1: New tools for the analysis of genome-wide SNP data. *Bioinformatics* **27**(21), 3070–3071 (2011). 21926124 10.1093/bioinformatics/btr521 PMC3198581

[32] Kamvar, Z. N., Tabima, J. F. & Grünwald, N. J. Poppr: An R package for genetic analysis of populations with clonal, partially clonal, and/or sexual reproduction. *PeerJ* **2**, e281 (2014). 24688859 10.7717/peerj.281 PMC3961149

[33] Minh, B. Q. et al. IQ-TREE 2: New models and efficient methods for phylogenetic inference in the genomic era. *Mol. Biol. Evol.* **37**(5), 1530–1534 (2020). 32011700 10.1093/molbev/msaa015 PMC7182206

[34] Paradis, E. & Schliep, K. Ape 5.0: An environment for modern phylogenetics and evolutionary analyses in R. *Bioinformatics* **35**(3), 526–528 (2019). 30016406 10.1093/bioinformatics/bty633

[35] Gómez-Gálvez, F. J. et al. New insights in the Spanish gene pool of olive (*Olea europaea* L.) preserved ex situ and in situ based on high-throughput molecular markers. *Front. Plant Sci.* **14**, 1267601 (2024). 38250447 10.3389/fpls.2023.1267601 PMC10796691

[36] Zunino, L. et al. Genomic evidence of genuine wild versus admixed olive populations evolving in the same natural environments in western Mediterranean Basin. *PLoS ONE* **19**(1), e0295043 (2024). 38232071 10.1371/journal.pone.0295043 PMC10793901

[37] Belaj, A. et al. Genetic diversity and population structure of wild olives from the north-western Mediterranean assessed by SSR markers. *Ann. Bot.* **100**(3), 449–458 (2007). 17613587 10.1093/aob/mcm132 PMC2533604

[38] Al-Kilani, M. A. et al. Evaluation of genetic diversity among olive trees (*Olea europaea* L.) from Jordan. *Front. Plant Sci.* **15**, 1437055 (2024). 39166249 10.3389/fpls.2024.1437055 PMC11333458

[39] Mariotti, R. et al. EST–SNP study of *Olea europaea* L. uncovers functional polymorphisms between cultivated and wild olives. *Genes* **11**(8), 916 (2020). 32785094 10.3390/genes11080916 PMC7465833

[40] Nteve, G.-M., Kostas, S., Polidoros, A. N., Madesis, P. & Nianiou-Obeidat, I. Adaptation mechanisms of olive tree under drought stress: The potential of modern omics approaches. *Agriculture* **14**(4), 579 (2024).

[41] Kaya, H. B. et al. Genome wide association study of 5 agronomic traits in olive (*Olea europaea* L.). *Sci. Rep.* **9**(1), 18764 (2019). 31822760 10.1038/s41598-019-55338-w PMC6904458

