# Supplementary material for: Genome-wide SNP analysis reveals genetic diversity and structure of wild and cultivated olives (Olea europaea L.) in Oman
Source: Sci Rep. 2026 Mar 1;16:11490. doi: 10.1038/s41598-026-40849-0 (PMC13057135; doi:10.1038/s41598-026-40849-0)
Supplement: Supplementary file 1 — Supplementary Information. [file 41598_2026_40849_MOESM1_ESM.docx]

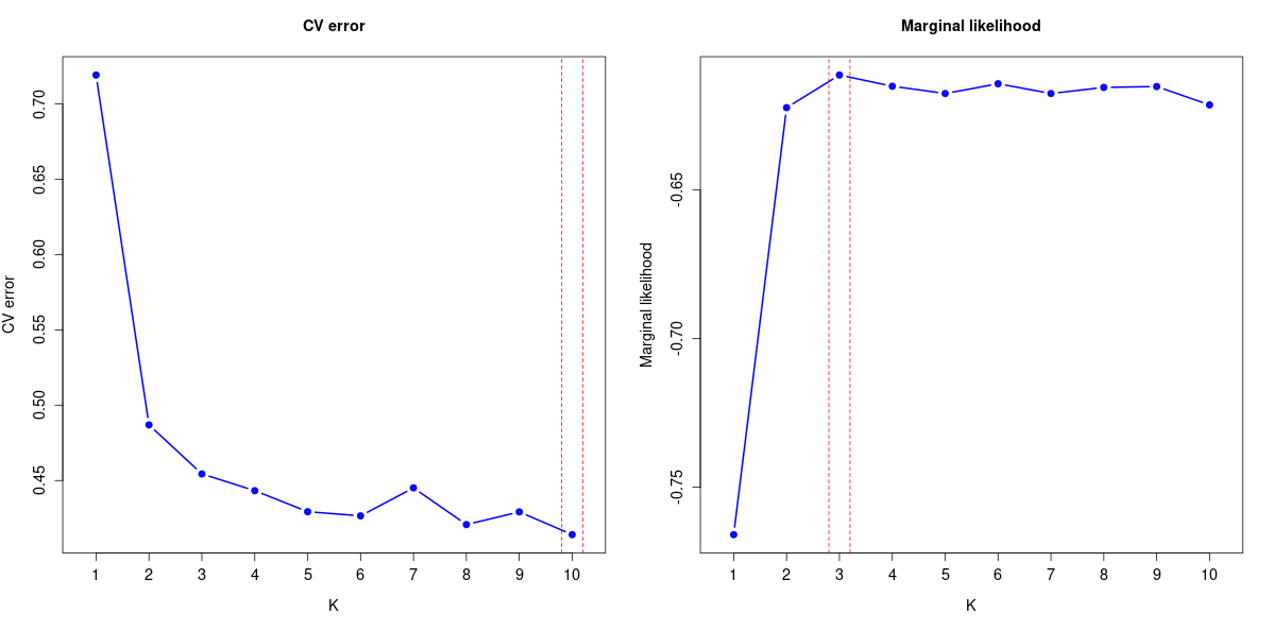


Supplementary Figure S1. Cross-validation error and marginal likelihood values across different numbers of genetic clusters (K) inferred using fastSTRUCTURE.


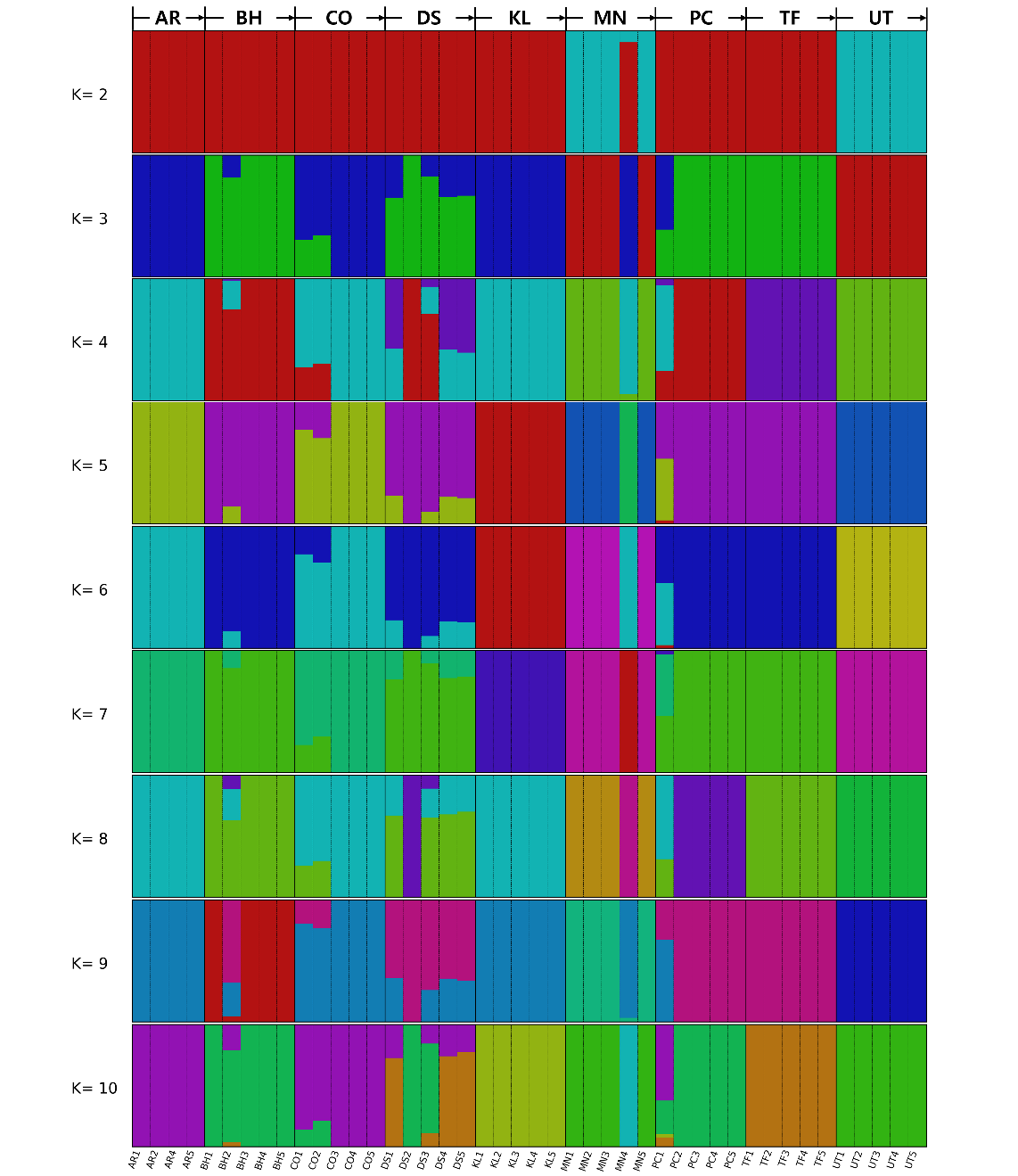


Supplementary Figure S2. Population genetic structure of 44 olive individuals inferred using fastSTRUCTURE across K values ranging from 2 to 10.
